# Analysis of voltage rise phenomena in electrical power network with high concentration of renewable distributed generations

**DOI:** 10.1038/s41598-022-11765-w

**Published:** 2022-05-12

**Authors:** Ayodeji Stephen Akinyemi, Kabeya Musasa, Innocent E. Davidson

**Affiliations:** Department of Electrical Power Engineering, Faculty of Engineering and the Built Environment, Durban University of Technology, Durban, 4000 South Africa

**Keywords:** Energy science and technology, Engineering

## Abstract

The increasing penetration levels of renewable distributed generation (RDG) into a power system have proven to bring both positive and negative impacts. The occurrence of under voltage at the far end of a conventional electrical distribution network (DN) may not raise concern anymore with RDGs integration into a power system. However, a penetration of RDGs into power system may cause problems such as voltage rise or over-voltage and reverse power flows at the Point of Common Coupling (PCC) between RDG and DN. This research paper presents the impact of voltage rise effect and reverse power flow constraint in power system with high concentration of RDG. The analysis is conducted on a sample DN, i.e., IEEE 13-bus test system, with RDG penetration by considering the most critical scenario such as low power demand in DN and a peak power injection by RDG. For studying the impact of voltage rise and reverse power flow, a mathematical model of a DN integrating RDG is developed. Furthermore, a controller incorporating an advance control-algorithm is proposed to be installed at PCC between DN and RDG to regulate the voltage rise effects and to mitigate the reverse power flow when operating at a worst critical scenario of minimum load and maximum generation from RDG. The proposed control strategy also mitigates the voltage–current harmonic distortions, improves the power factor, and maintain the voltage stability at PCC. The simulations are carried out using MATLAB/Simulink software. Finally, recommendations are provided for the power producers to counteract the effects of voltage rise at PCC. The study has demonstrated that, voltage at PCC can be sustained with a high concentration of RDG during a worst-case scenario without a reverse power flow and voltage rise beyond grid code limits.

## Introduction

The integration of RDG into the power system is ever increasing on daily basis which can be ascribable to the policy regulation of energy because of global warming, increasing environmental concerns of the adverse effect of fossil fuel usage, regular outage and blackout, inadequate access to the utility grid by the rural communities and the high price of electricity billing^1–3^. The conventional power system has been intrinsically radial, i.e., power flows in one direction only: from power plant to transmission network, distribution network, and to the loads. These flows are traditionally managed through the dispatch of generation and network equipment such as tap-changing transformers that can adjust network voltages, i.e., the voltage settings at the last controllable transformer before the loads are often set at 5–10% higher than the nominal end-use voltage to accommodate line losses and voltage drops along the line. These losses and associated voltage drop depend, of course, on the actual current flows that are being demanded by the loads. However, the introduction of RDG changes the dynamic of the network because power flows may change significantly and potentially in both directions. In other words, the network becomes an active system with power flows and voltages determined by the mix of centralized power, RDG and as well as the load. Thus, with significant increase in penetration level of a large RDG, over voltage will occur at PCC, the voltage at the load end would be greater than the feeder supply voltage, this is called the voltage rise and can also cause the voltage to flow back to the feeder supply side known as reverse power flow. The reverse power flows and voltage rise are worsened when customer demand is at its lowest and RDG supply is at its highest, such issue would be critical especially on the long feeders such as in rural areas. Repeated switching of RDG systems on and off or complete disconnection of RDG from the grid and other methods suggested in Refs.^4–14^ in response to voltage rise and the reverse power flow issues could only provide a temporary measure. Consequently, the resultant effects can impose consequent cycling of network voltage control equipment with associated asset life and maintenance impacts, can cause partial/total outages or excessive under voltages at the far end of the DN, damaging online power component, end-use equipment and voltage instability^15–17^. Hence, permanent solution is required.

Similarly, there are several technical issues reported by distribution system operators (DSO) that are associated with the increase in penetration level of RDG integration such as voltage rise, voltage instability during grid disturbance, power flowing back to the substation, voltage–current harmonic signals, etc.^18^. These challenges may persist for a long period of time if DSO does not have a foreknowledge of the power flow with RDG integration, which can cause serious damage to the power system components/equipment and the load connected to the system. For RDG penetration level to be sustained without disconnection from the system, an over voltage at PCC must be managed. The power flows and the voltage profiles of a traditional electrical distribution system cannot remain the same when considerable amounts of renewable energy-based distributed generation are integrated. Typical RDGs inject active power into the grid, increase in penetration level may cause an over-voltage/voltage rise at the PCC which must be regulated as specified by IEEE 1547 if the facilities are to continue operation.

The operating condition of DN would be vital when considering a large RDGs with increasing penetration level due to the potential voltage rise and the reverse power flow threat at PCC. The PCC voltage rises at the critical scenario of low load and peak RDG generation, such that the depth of voltage rise depends on the injection of active and the reactive power from the RDG. The bidirectional flow of energy from the RDG as well as the main utility grid causes several difficulties regarding the DN voltage profile, power quality, security, power flow control, energy management, frequency control and protection. Network protection and security when a large RDG is connected to DN has been a significant concern in recent years and needs an urgent attention. The RDG injects active and the reactive power to the grid at the PCC. Thus, PCC point is more active than other nodes in the system, often the PCC’s voltage is higher than other Busbar voltages within the network. The more the PCC is becoming an active point due to the increase in the RDG penetration level, there would be a voltage rise and the reverse power flow threat at the PCC. The voltage rise beyond the required limit set by the utility will occur at the critical scenario. At this critical stage, it is either RDG is disconnected from the network to avoid damage to other facilities within the system or the voltage rise and the reverse power flow occurrence are regulated to an acceptable range. The unprecedented behaviour of the feeder due to the impact of a large RDG system has drawn keen interest of researchers worldwide and it has resulted in the development of analytical tools for investigating these impacts to develop mitigation measures to curb some of the issues and challenges on distribution feeders.

A considerable number of research efforts have been carried out in the literature to assess the impact of voltage rise with RDG integration and to provide grid support among different voltage controls. In most of the foregoing literature, the researchers have only focused on generation curtailment in DN with RDG integration. Nevertheless, a few studies have been explored to alleviate voltage violation. Reference^19^ established that voltage rise depends on the value of line resistance and real power injection. With the advent of over generation of power from RDG, there may be a need to curtail some RDG real power or absorb reactive power by RDG inverters to eliminate the voltage rise. It is also established that highest voltage rise may occur at the farthest bus after the RDG integration in the DN. It is also observed in Ref.^20^ that with an increase in the RDG penetration level to DN, the voltage rise increases, and this increase in the voltage rise is attributed to the impedance of the line. Reference^21^ proposed phase shifting strategy (PSS) for mitigation of the voltage rises and reverse power flow. On-Line-Tap-Changer (OLTC) in conjunction with D-STATCOM were used to mitigate the voltage rise in DN^22,23^ while^24^ proposed two D-STATCOM and OLTC for voltage regulation and to mitigate voltage unbalance in DN. Reference^25^ utilized dynamic voltage restorer (DVR) that utilizes a matrix converter to mitigate power quality problems such as voltage dip, swell and obtained the compensation energy directly from the power system. References^26,27^ established that there would be a reverse directionality of the power flow to the electric grid with RDG integration and low load consumption. The reverse power flow constraint at PCC caused by a high concentration of RDGs and a minimum power demand in DN were also buttressed in the literature review with mathematical analysis; and it has been demonstrated that, increasing of power output from RDGs will inevitably cause a rising of voltage at PCC. This voltage rise may spread throughout the feeder/or source in case more RDGs are connected to the main knot of the feeder. It is also suggested in Ref.^28^ that the mitigation of voltage rise can be achieved by controlling the active and reactive powers using a voltage source converter.

The performance of the voltage rise during transient faults when RDG sources are integrated to DN has been consistently investigated. The ability of RDGs to stay connected to the grid during fault conditions for a specified period is referred to as low voltage ride-through (LVRT) ability in the literature reviewed^29,30^. Various advanced techniques have been provided by the researchers to improve the RDG ride-through capability where most of the control strategies adopted techniques worked in normal operation mode and under a fault condition^31^. The response of a RDG to a short circuit fault depends on the type of renewable generators employed as described in Refs.^32,33^, if a fault is applied at the terminal of a RDG, the short circuit current can reach up to five to six per unit (p.u.) of the rated voltage/current that could damage the generator. Reference^34^ recommended an approach to enhance the low voltage ride-through ability based on inductance-simulating control to coordinate voltage/current which minimized the post-fault current within the acceptable range. Reference^35^ suggested an improved fault ride-through for a RDG under symmetrical and unsymmetrical faults, the scheme trusted with the control of the inverter and grid side converters to decrease overcurrent and inject reactive power to the grid side to support voltage recovery. References^36,37^ developed an optimum resistive type of fault current limiter to achieve maximum fault ride-through capability with RDG which is an effective control strategy for a low voltage ride through for RDG. In addition, increase in the penetration level of the RDG to the DN can cause transient overvoltage during rapid earth fault which may lead to wildfire. It may also trigger some sensitive overvoltage protective devices. The effects could be mitigated by the voltage clamping-based overvoltage protection strategy that has proposed and developed through a smart power electronic topology^38^. Moreover, the previous studies have not conducted a comprehensive analysis of voltage rise effects and reverse power flow with RDG integration at the PCC of a DN in alleviating voltage rise concerns. However, due to the rising number of RDG installations in the DN, new voltage management approaches are required to be adopted to mitigate the voltage rise concerns. This paper presents the analysis of voltage rise effects with RDG integration at the PCC of a DN. The following are the research questions that guide the study:

- Can high concentration of RDGs integration cause a voltage rise on a distribution network?
- What effect or impact will the RDGs have on the reverse power flow into a distribution network?

If there is adequate information and understanding of how RDG integration and penetration level will appear in a distribution network as this will expand the monitoring efforts of DSO to operate the grid efficiently and economically in reaction to the evolving challenges of RDG penetrations into a DN. The contribution of this research paper is as follows:

- This study develops a mathematical model of an electrical distribution network with an RDG—connected. The developed model is further used to investigate the effect of voltage rise on an electrical distribution network.
- To design an advance controller with PWM control-algorithm to normalize the voltage rise at PCC and to mitigate the reverse power flow when operating at a worst critical scenario of minimum power demand and maximum power output from RDG.
- The installation of PWM based DSTATCOM at PCC for the control of active and reactive power.

The solutions developed in this research paper can be useful for DSOs and independent power producers at the planning and installation stage of RDG integration. The paper is structured in three sections. The first section is the introduction while the second section discusses the methods which comprises of the power system design and RDG, Mathematical model derivation for the analysis of voltage rise effects and the description of the test system under investigation. The last section presents the results and discussion which is divided into subheadings, such as the simulation results, voltage rise concept, the impact of RDG on a distribution network, RDG integration versus voltage rise at PCC, the voltage rise compensation methods, RDG integration and voltage rise at PCC, voltage rise compensation methods, compensator connection model, signal analysis, small signal analysis and voltage rise regulation, simulation analysis are presented while eight summarizes the conclusion.

## Methods

### Power system design and renewable distributed generation

**Figure 1:**
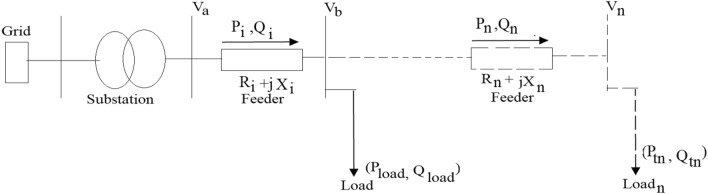
Equivalent circuit of an electrical distribution system.

In the traditional or conventional electrical distribution networks, generally power flows from the substations to the loads in a unidirectional manner as shown in Fig. 1. Integration of RDGs into the power system has changed the features of the power system; actually, power flows change from unidirectional to bidirectional. Table 1 provides a summary of changes in power system with RDG integration. The active and the reactive power usually flow from higher voltage potential to the lower voltage level, the ratio of reactance to the resistance of the distribution network is also less than or equal to (1/2), while that of the transmission network is greater than or equal 10. Consequently, the value of the resistances in the transmission network is lower as compared to the distribution network, this high resistance in the distribution network is responsible for the voltage drop along the feeders from the sending end to the receiving loads.

**Table 1:** Power system design and RDG.

Comparison between conventional power system and RDG design		
S/N	Conventional	RDG
1	Unidirectional power flow	Bidirectional power flow
2	Centralized generation	Distributed generation
3	Manual monitoring	Self-monitoring
4	Manual restoration	Automatic restoration
5	One-way communications	Two-way communications
6	Electromechanical	Digital
7	Few sensors	More sensors
8	Limited control	Full control
9	Failures and blackouts	Adaptive and resilient

### Mathematical model derivation for voltage rise analysis

Considering an electrical distribution network in Fig. 1 to analyze the voltage limit violation challenges. This feeder comprised of *n* buses, and on each bus, loads are connected. The voltage differential between two consecutive buses depends on the line segment impedance and the active and reactive power flows as deduced in Eq. (1). Where V_a_ and V_b_ is the sending end terminal voltage and the receiving end terminal voltage as indicated in the Abbreviation section.

1 $${V}_{b-a}-{V}_{b}\cong \frac{{R}_{i}{P}_{i}+{jX}_{i}{Q}_{i}}{{V}_{b}\cong 1 p.u.},$$

where ${P}_{i}$ and ${Q}_{i}$ are the active and reactive power flow through the ${i}_{th}$ section of the line which depend on the active and reactive power absorbed in the feeder buses. When power losses are neglected, Eqs. (2) and (3) can be deduced.

2 $${P}_{b}\cong {P}_{b-a}-{P}_{{t}_{b-a}},$$

3 $${Q}_{b}\cong {Q}_{b-a}-{Q}_{{t}_{b-a}},$$

where ${P}_{{t}_{b}}$ and ${Q}_{{t}_{b}}$ are the active and reactive power absorbed in the ${i}_{th}$ bus, which are equal to the difference between the power consumption of the load (${P}_{Load}\, {\text{and}}\, {Q}_{Load}$) and the output power of the RDG (${P}_{RDG}\, {\text{and}}\, {Q}_{RDG}$) connected to this bus is deduced in Eqs. (3) and (4).

4 $${P}_{{t}_{b}}\cong {P}_{Load}-{P}_{RDG},$$

5 $${Q}_{{t}_{b}}\cong {Q}_{Load}-{Q}_{RDG}.$$

From the Eq. (1), the voltage difference between the ${i}_{th}$ bus and the main substation bus can be expressed in Eq. (6).

6 $$\begin{aligned} V_{sub} - V_{b} =\, & \mathop \sum \limits_{j = 1}^{b} R_{j} P_{j} + X_{j} Q_{j} = \mathop \sum \limits_{j = 1}^{b} R_{j} \left( {\mathop \sum \limits_{k = j}^{n} P_{{t_{k} }} } \right) + X_{j} \left( {\mathop \sum \limits_{k = j}^{n} Q_{{t_{k} }} } \right) \\ = \,& R_{1} \left( {P_{{t_{1} }} + \cdots + P_{{t_{n} }} } \right) + X_{1} \left( {Q_{{t_{1} }} + \cdots + Q_{{t_{n} }} } \right) + \cdots + R_{b} \left( {P_{{t_{b} }} + \cdots + P_{{t_{n} }} } \right) + X_{b} \left( {Q_{{t_{b} }} + \cdots + Q_{{t_{n} }} } \right). \\ \end{aligned}$$

**Figure 2:**
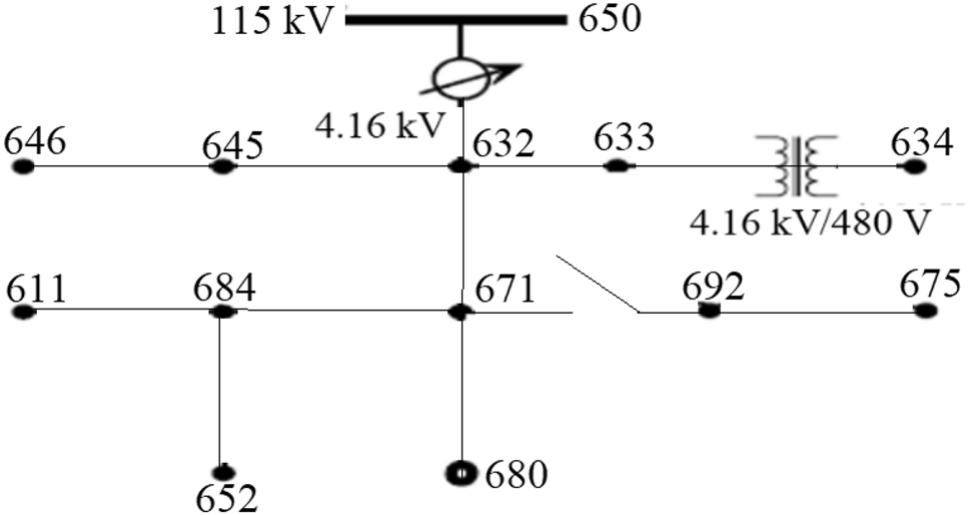
IEEE 13 Node feeder test system.

From Eq. (6), when there is no RDGs, the power absorbed by the load increased, then the internal bus voltage decreased relative to the sending end voltage bus. This can cause a voltage drop across the network end buses from its allowable range under high demand scenario. However, with an increase in the penetration level of RDG in the system, during peak generation hours of RDGs and low demand scenario, the power absorbed on each bus reduces resulting in the reverse power flow through the feeder. In this condition, ${P}_{{t}_{b}}$ becomes negative, and the internal bus voltage increases relative to the sending end voltage resulting in voltage rise occurrence. Conversely, depending on the location of power absorption or injection in the network, its impact on the system voltage profile is not the same. It is very clear from Eq. (6) that the active and the reactive power injected at the end buses flow through the larger impedance which can create more substantial consequence on the voltage of these terminals. Change in voltage can be expressed in per-unit or percentage and by considering V_a_ and S_a_ as base parameters in Eq. (7).

7 $$\Delta V\approx RP+XQ,$$

where, P and Q are the per-unit active and reactive power; and R and X are the per-unit values of the line impedance. The voltage variation limit at the PCC on demand or consumer sides is very vital for power quality as specified by IEEE 1547 std. The IEEE standard grid code allows a ± 6 voltage variation at PCC when connecting RDG to an electrical network, while the South Africa grid code regulation allows − 15% to + 10% (0.85 to 1.1 voltages per unit) for low voltage and ± 10% for medium and high voltage around the nominal value^39^. The voltage level at the point of load connection can be investigated using the standard IEEE 13 Node feeder test system^40,41^ in Fig. 2.

### Test system description

Modified IEEE 13-bus test system, shown in Fig. 2 is used in this research paper to investigate the impacts of RDG integration to an electrical distribution network and also to test the performance of the controller for voltage rise regulation. The IEEE 13-bus test system is a sample standard distribution network for voltage profile analysis. It is comprised of both the medium and the low voltage distribution network interconnected via a three-phase 4.16 kV/480 V transformer. This model was developed by the IEEE Power Engineering Society’s Power System Analysis, Computing and Economics Committee^42,43^. The test system is modified and modelled as balanced three phase in the MATLAB/Simulink environment; all the underground lines are modelled as overhead lines because there is no underground cable in the Simpower system’s library of Simulink. The network loading is modified to be 480 kVA to 1 MVA while the capacity of the distribution station is 510 kVA with 4.16 kV voltage level, the total length of the network is 25 km. The detail parameters of the system are depicted in “Appendix”.

## Results and discussion

### Test system simulation

**Figure 3:**
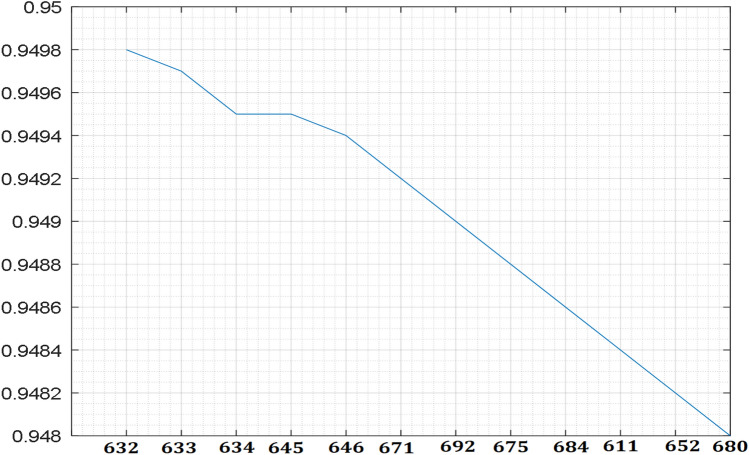
Test system voltage profile.

The test system is simulated, the measured bus voltage and the graphical representation of the measured voltage parameters obtained from the system are shown in Table 2 and Fig. 3. The network allowable voltage variations are guided by the IEEE 1547 which allow ± 6 variation in PCC voltage and South Africa grid code that allows − 15% to + 10% (0.85 to 1.1 voltages per unit) for low voltage and ± 10% for medium and high voltage around nominal voltage^39,44^. It is observed that the system voltage profile variations are within the specified limit of both ± 6 and − 15% to + 10%. However, if the distance of the distribution line in Fig. 1 is assumed to be longer than 25 km and the network loading is greater than the current loading, by analogue thinking, the farthest nodes such as node 652 or 680 of the voltage profile in Fig. 3 will fall outside the specified voltage limit. Hence, utility company usually runs their networks to be within the allowable voltage ranges and regulates the network voltage with the aid of automatic voltage regulator and on tap changing transformers.

**Table 2:** Base case measured voltage.

Bus no	Voltage (pu)
632	0.9498
633	0.9497
634	0.9495
645	0.9495
646	0.9494
671	0.9492
692	0.9490
675	0.9488
684	0.9486
611	0.9484
652	0.9482
680	0.9480

### Voltage rise concept

The conventional distribution networks usually designed to have a stable voltage profile initially such that the voltage rise may not be the concern^2^ but under voltage, meanwhile, when RDGs are integrated into the network, the power flow is no more unidirectional such that power flows is not only from the substation to the farthest node of the system anymore, but the power can also now flow back from the farthest node towards the substation due to the RDG integration. The system voltage profile would definitely be affected by the RGDs integration because the network is no more passive but has become an active network. The voltage generated by the RDGs must be higher as compared to the voltage of the other nodes around the PCC for the power to be exported to another part of the network. This can be best described by the Eq. (7) and the equation parameters are described in the Abbreviation section. Therefore, the receiving end voltage ${(V}_{b})$ can be expressed in Eq. (8).

8 $${V}_{b}\approx {V}_{a}+RP+XQ.$$

**Figure 4:**
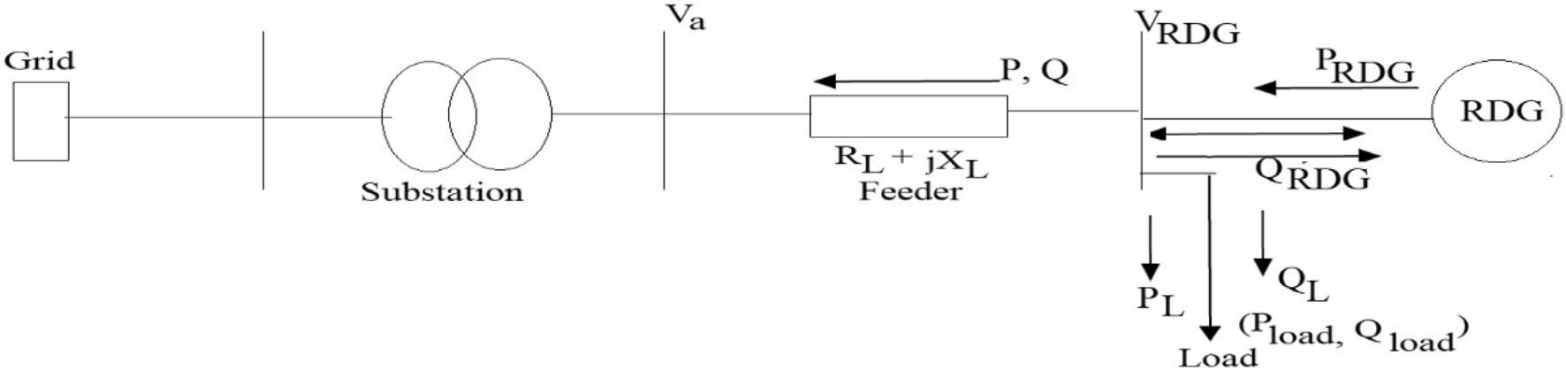
RDG integration to distribution network.

The change in the power flow direction because of RDG integration resulted to the generated voltage at PCC to rise above the sending end voltage such that the node at which RDG is connected to a distribution network will form active point, the weak node will become an active node, further increase in the number of RDGs and their penetration levels will also make the nodes near the PCC to be more active resulting to fewer weak zones in the system. Hence, active zones become smart zone, further increase in RDGs penetration levels will eventually result in a sufficient smart point to control the whole local network. The more the PCC is becoming active point, there is a potential voltage rise threat at that point if the voltage at that point is not regulated which can be analysed in Fig. 4.

RDG is integrated into a distribution network via the distribution line with impedance as shown in Fig. 4, the nomenclature of the circuit equations is defined in the nomenclature table. The rise in voltage at PCC due to the RDG integration can be expressed in Eq. (9).

9 $$\Delta V={V}_{RDG}-{V}_{a}\approx \frac{RP+XQ}{{V}_{RDG}},$$

where $$P={(P}_{RDG}-{P}_{L})$$ and $$Q=(-{Q}_{L}\,\pm\, {Q}_{RDG}$$).

From Eq. (9), ${V}_{GEN}$ can be expressed in term of per unit in Eq. (10).

10 $$\Delta V={V}_{RDG}-{V}_{a}\approx {R(P}_{RDG}-{P}_{L})+X(-{Q}_{L}\,\pm\, {Q}_{RDG}).$$

Most of the time, RDG exports active power $(+{P}_{G})$ to the grid and the reactive power $(\,\pm\, {Q}_{G})$ can also be exported or imported from/or to the grid while the load active and the reactive power $({P}_{L} \text{ and } {Q}_{L})$ are being consumed by the loads. Depending on the type of RDG that is integrated into the distribution network, some export real power to the grid when the loads connected to the system reduce below the generator output, whereas reactive power could be exported or absorbed depending on the settings of excitation scheme of the RDG such as synchronous generator when it is use for wind energy converter while induction generator consumes reactive power to operate. Solar Photovoltaic exports real power to the grid at a predetermined power factor, the flow of power could occur in both directions based on the real and the reactive power loading of the system as compared to the output of the generator and the system losses.

### Impact of RDG on distribution network

**Figure 5:**
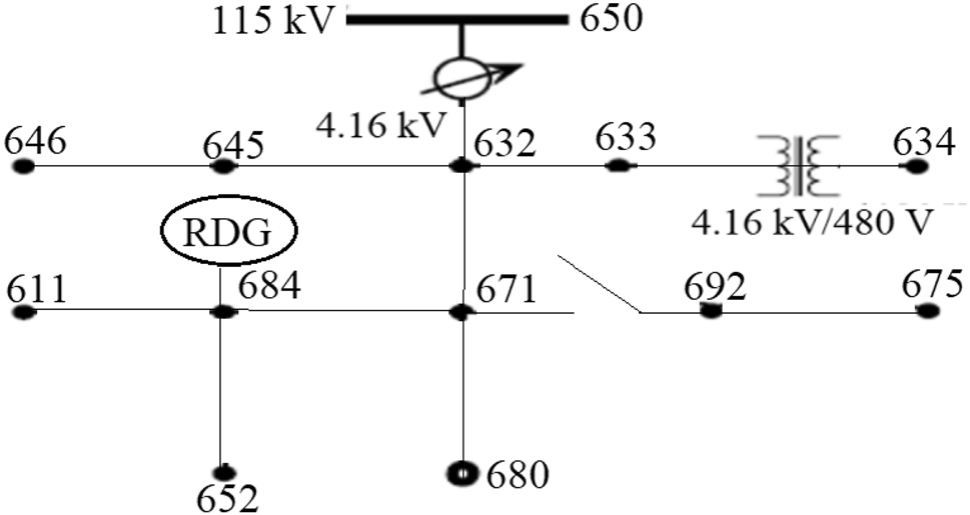
RDG connection to distribution network.

**Figure 6:**
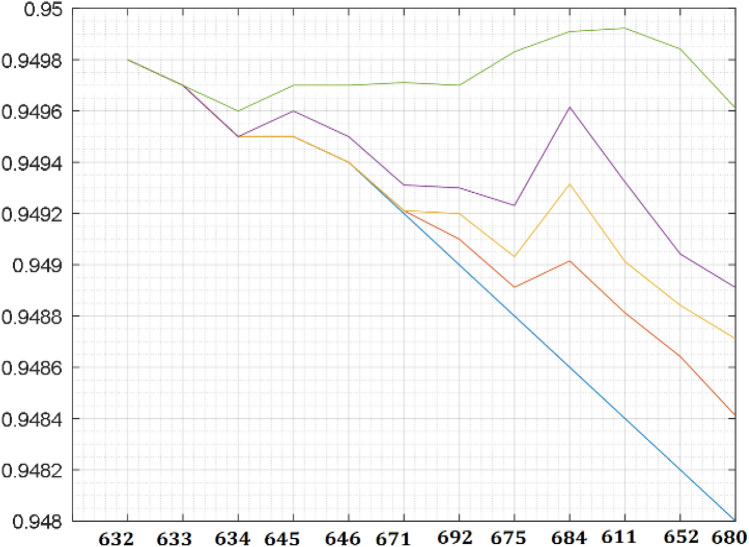
Improved DN voltage profiles at (10–40%).

**Figure 7:**
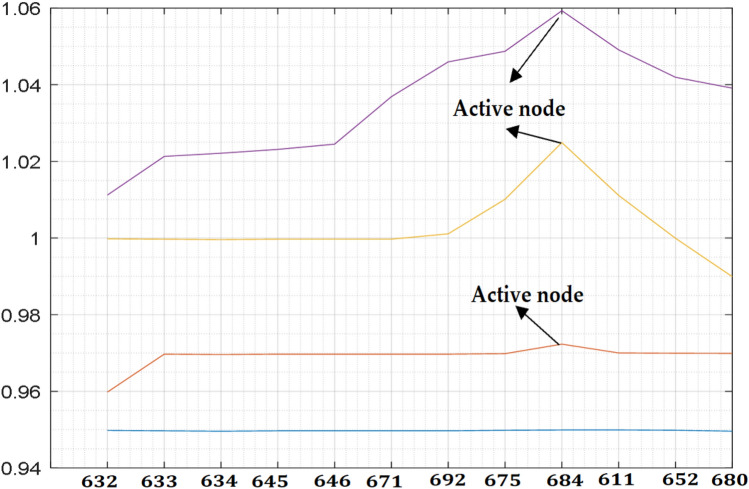
Improved DN voltage profiles at (50–80%).

This section investigates the impact of RDG on a distribution network. A RDG of 240 kW with a unity power factor is integrated into the network of Fig. 2 as shown in Fig. 5 to meet a certain customer load demand while the distribution substation voltage is controlled at 100%. Tables 3 and 4 depict the measured voltage values from each node of the network while Figs. 6 and 7 depict the graphical representation analysis of the simulation results. It is observed that the network voltage profiles improved considerably with the increase in the RDG integration penetration level and is within an acceptable voltage range. It is also observed that the voltage of the node 684 at which the RDG is connected (PCC) is higher than any other voltages of the network as previously said as shown in Figs. 6 and 7, this is because of the injection of active power by the RDG which makes that particular bus voltage (PCC) to be active than any other node, the impacts are noticeable at the PCC and the closest node in both directions around the PCC. The voltage profiles are within an acceptable range as specified by IEEE 1547 and the South Africa grid code act of RDG connection at PCC. The investigation of the impacts of RDG integration into power system based on the research investigation and the simulation carries out with the results obtained in this section implies that the impact of RDG integration will improve the distribution network voltage profiles and make the weak node/bus/network active. Hence, a weak node can become an active node with RDG integration while a weak network can become an active network. With the voltage profiles improvement with RDG integration as established in the results (Tables 3, 4, Figs. 6, 7) of the investigation carried out in this paper, the first research question is satisfied such that the impact of RDG with an increase in penetration levels can be noticeable in the power system.

**Table 3:** Measured voltage at (10–40%) RDG.

Bus no	Base Voltage	10%	20%	30%	40%
632	0.9498	0.9498	0.9498	0.9498	0.9498
633	0.9497	0.9497	0.9497	0.9497	0.9497
634	0.9495	0.9495	0.9495	0.9495	0.9496
645	0.9495	0.9495	0.9495	0.9496	0.9497
646	0.9494	0.9494	0.9494	0.9495	0.9497
671	0.9492	0.9492	0.9492	0.9493	0.9497
692	0.9490	0.9491	0.9492	0.9493	0.9497
675	0.9488	0.9489	0.9490	0.9492	0.9498
684	0.9486	0.9490	0.9493	0.9496	0.9499
611	0.9484	0.9488	0.9490	0.9493	0.9499
652	0.9482	0.9486	0.9488	0.9490	0.9498
680	0.9480	0.9484	0.9487	0.9489	0.9496

**Table 4:** Measured voltage at (50–80%) RDG penetration.

Bus no	50%	60%	70%	80%
632	0.9498	0.9598	0.9998	1.0112
633	0.9497	0.9697	0.9997	1.0212
634	0.9496	0.9696	0.9996	1.0221
645	0.9497	0.9697	0.9997	1.0231
646	0.9497	0.9697	0.9997	1.0245
671	0.9497	0.9697	0.9997	1.0368
692	0.9497	0.9697	1.0011	1.0459
675	0.9498	0.9698	1.0101	1.0487
684	0.9499	0.9723	1.0249	1.0593
611	0.9499	0.9700	1.0111	1.0491
652	0.9498	0.9699	0.9999	1.0419
680	0.9496	0.9699	0.9899	1.0391

### RDG integration and voltage rise at PCC

**Figure 8:**
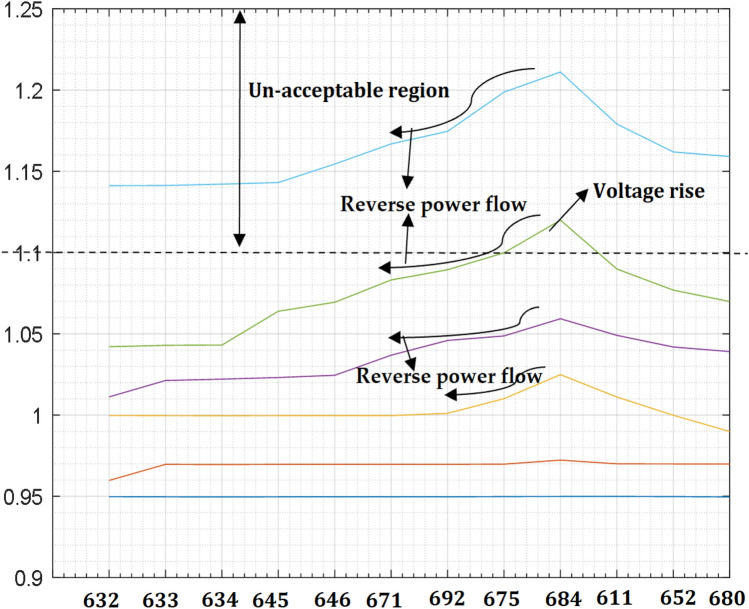
Improved DN voltage profiles at (60–100%).

The network loading remains the same while penetration levels of RDG into the network increases from 240 kW at bus 684 to 1 MW, the buses measured voltages are depicted in Table 5 and the graphical analysis of the simulation are shown in Fig. 8. The increase in the RDG penetration levels causes reverse power flow towards the distribution substation as depicted in Fig. 8 around the nodes 675 and 692. The voltage at bus 684 (PCC) rises above the maximum allowable voltage range of 1.1 pu due to the integration of a large RDG. The over voltage occurs in both directions such that the power flows towards the distribution substation (see Figs. 7, 8, buses 675 and 692) and also to the farthest bus of the network (see Figs. 7, 8, buses 611 and 652). Although, the technical performance of RDG integration can make a weak distribution network to be an active network nevertheless, the impacts of a large RDG can a cause voltage rise at PCC and reverse power flows which satisfied the second research question. By analogous thinking, similar occurrence may occur during low load and high generation of RDG integration. It is therefore worthy to note that before considering RDG for an integration into the power system, the power system operator should consider the possibility of power being exported back to the substation in-case there is an over generation of power from RDG as this will give them the ideal of choice of transformer to be installed that can tolerate the operation of reverse power flow.

**Table 5:** Measured voltage at 1 MW penetration level.

Bus no	60%	70%	80%	90%	100%
632	0.9598	0.9998	1.0112	1.0420	1.1412
633	0.9697	0.9997	1.0212	1.042	1.1412
634	0.9696	0.9996	1.0221	1.0431	1.1421
645	0.9697	0.9997	1.0231	1.0639	1.1431
646	0.9697	0.9997	1.0245	1.0694	1.1545
671	0.9697	0.9997	1.0368	1.0831	1.1668
692	0.9697	1.0011	1.0459	1.0894	1.1745
675	0.9698	1.0101	1.0487	1.0998	1.1987
684	0.9723	1.0249	1.0593	1.1200	1.2111
611	0.9700	1.0111	1.0491	1.0899	1.1791
652	0.9699	0.9999	1.0419	1.0769	1.1619
680	0.9699	0.9899	1.0391	1.0699	1.1591

The simulation in Fig. 8 satisfies the Eq. (10) such that RDG exports active power $(+{P}_{G})$ to the grid. Hence, with RDG integration, the threat that an under-voltage will occur at the far end of the system cannot exist again.

The power system deregulation, reliable power supply, power quality, meeting customers’ load demand, economic value and the environmental regulation of greenhouse emissions are some of the primary aims of deploying RGDs integrating into the power system which is seen fulfilling these purposes. Therefore, Eq. (10) can be further expressed in Eq. (11)

11 $${P}_{G}\approx \frac{{P}_{RDG}-{V}_{a}+R{P}_{L}-X\left(-{Q}_{L}\,\pm\, {Q}_{RDG}\right)}{R}.$$

Thus, from the Eq. (11), the level of RDG that can be integrated into a distribution network can be deduced which depend on the following:

- The voltage at the distribution substation.
- The voltage level at the farthest bus.
- The distance of the network and the conductor size.
- Load demand within the network.

There are critical situations that can be investigated and put the outcome into consideration when RDGs are to be integrated into a distribution network to regulate the activities of RDGs at the PCC especially the impacts on the voltage rise such as:

- Peak load and Peak RDG generation.
- Peak load and low RGD generation.
- Low load and Peak RDG generation.
- No load and Peak RDG generation.

If the most critical situation is to be considered such that there is a reduction in the load demanded with a peak RGD generation, the analysis of such situation can be expressed by employing Eq. (10) and re-express in Eq. (12). Meanwhile, if the network is operating at unity power factor, then the Eq. (13) is valid. Based on this assumption, the Eq. (10) can be re-expressed in Eq. (14).

12 $${P}_{L}=0, {Q}_{L}=0, \,{\text{and}}\, {P}_{RDG}={P}_{RDG\, Max},$$

13 $$\pm\, {Q}_{RDG}=0,$$

14 $$\Delta {V}_{Critical}={V}_{RDG\, Max}-{V}_{a}\approx R{P}_{RDG\, Max}.$$

From the Eq. (14), it can be observed that the PCC voltage rise depends on the resistance (R) of the distribution lines and the penetration power of the RDG. Hence, if the resistance of the distribution line remains unchanged, the Eq. (14) can be re-expressed in Eq. (15), from the Eq. (15), it can be deduced that the amount of voltage in a distribution network with RDGs integration is directly proportional to the active power injected into the network by the RDGs.

15 $$\Delta {V}_{Critical} \,\alpha\, {P}_{RDG\, Max}.$$

A linear relationship exists between the active power generated by the RDGs and the occurrence of voltage rise at PCC. The voltage rise would be burdensome and raise a concern when there is no load demand in the network due to the fact that the power injected by the RDG would be exported back into the distribution substation and if such thing happens, damaging of power system equipment and components are inevitable. Furthermore, the occurrence of voltage rise at PCC because of impacts of RDGs penetration levels can limit the amount and the extent at which RDG can be integrated into the power system. The above statement can be proved from the Eq. (13), which can be re-expressed in Eq. (16).

16 $${P}_{RDG\, Max}=\frac{{V}_{RDG\, Max}-{V}_{a}}{R}.$$

The amount of RDG that can be integrated into the existing network would be limited by the peak/maximum voltage produced by the RDG connected to PCC which is expressed in Eq. (17).

17 $${P}_{RDG\, Max}\le \frac{{V}_{RDG\, Max}-{V}_{a}}{R}.$$

Hence, from the critical situation, it can be observed that the resistance of the distribution line and the voltage rise at the network nodes is vital to the amount of RDGs penetration to the distribution network.

### Voltage rise compensation methods

With RDG integration into the distribution network, the voltage level of the system will be altered, and power flows will now be bidirectional. With the integration of RDGs into the power system, the voltage sag may not be the foremost concern anymore since active power injected by the RDG will cause the system voltage to increase. Hence, RDG integration introduces a new challenge, the voltage at the Point of Common Coupling (PCC) of RDG is higher as compared to the other buses of the network. Hence, active power increases with an increased penetration level. This results in the voltage increase at the PCC, thereby causes a voltage rise. Voltage rise challenges were reported as the foremost concern against the connection of RDG to medium and low-voltage distribution networks^45–48^. A large RDGs integration into a distribution network can cause an extreme voltage rise at PCC if not regulated or control appropriately. Conventionally, most of the distribution substations are fortified with an automatic over-voltage protection strategy to safeguard the power system equipment, component and loads of excessive voltage rise^49^. Nevertheless, sometimes the protection scheme arrangement can disconnect RDG permanently from the network or can also disconnect distribution supply from the main grid which can have a critical effect on the customer loads connected to the system and independent power producers causing loss of revenue. The voltage rises at the PCC with RDG integration can therefore be regulated through the following strategies which can be applied at the planning and execution stage of RDG integration by the utility or independent power producers in the power system.

- Reduction in the distribution resistance.
- Distribution substation Voltage control method.
- RDG penetration curtailment.
- Reactive power compensation strategy.

### Distribution line resistance reduction

The voltage rise at the PCC poses a great limitation of a large RDG integration into a distribution network. Since voltage is directly proportional to the current flowing through a resistance (Ohm’s law), which means voltage increases as current or resistance increases. For alternating current network, the impedance comprises of inductance, resistance and capacitance, by adjustment of these components, voltage increases or decreases. Thus, the voltage drops as a result of the impedance of the feeder, the flow of the current, the load, the transformer and the source voltage define the voltage at the end of the feeder. If the amount of RDG integration into the Distribution network is remained unchanged, thus, Eq. (18) can be deduced from Eq. (16).

18 $$\Delta {V}_{Critical}\, \alpha\, R.$$

The Eq. (18) shows the critical situation whereby the voltage rise from RGD peak penetration is directly proportional to the distribution line resistance. Hence, by reducing the line resistance the voltage rise would be reduced considerably. This process can be logically carried out by increasing the size of the conductors of the distribution network. This method may be slightly difficult to implement on the existing distribution system, but it can be proposed and implemented in the new distribution network. It is therefore recommended for the utility company to put into consideration the reduction in the distribution line resistance by the increasing the conductor size while constructing a new distribution system as this will enable a large RDGs integration to the system.

### Distribution substation voltage control method

In a conventional distribution system, it is usually a standard to sustain distribution substation voltage above nominal voltage value. This process is carried out to keep the network voltage within an acceptable range as specified by the IEEE 1547 and South Africa connection act ($\,\pm\, 6$ or 0.85 to 1.1 pu). However, this situation is not valid when RGDs are integrated into the system as investigated and confirmed in the simulation result in Fig. 8. From the Eq. (19), if the voltage supply from the distribution substation can be controlled, then the voltage drop can be regulated.

19 $${\Delta V}_{Critical}={V}_{RDG\, Max}-{V}_{a}.$$

This method of controlling the voltage supply from the distribution substation can easily be carried out using Online Tap Changing Transformer (OLTCT). The voltage regulation is possible in this regard in a short distribution network however, such practice may be cumbersome in a long-distance distribution network because there exist more transformers in a long distribution system, carry out such a practice may not be practicable. Nevertheless, by optimising the supply voltage value and online tap changing transformer tap position, the system voltage can be regulated to the minimum.

### RDG penetration level curtailment

The occurrence of the voltage rises at the PCC because of the integration of large RGDs into a distribution can be regulated through the RDGs integration penetration levels curtailment. The consequence of RDGs curtailment can be expressed in Eq. (20), this equation can be re-expressed in Eq. (21).

20 $${P}_{RDG\, Max}\approx {P}_{RDG\, Curtailment}+\frac{{V}_{RDG\, Max-{V}_{a}}}{R},$$

21 $$\Delta {V}_{Critical}\approx {RP}_{RDG\, Max}-{RP}_{RDG\, Curtailment}.$$

From the Eq. (21), it can be observed that by curtailing the RDG integration penetration levels, voltage rise can be therefore regulated at PCC. The critical situation is not always occurred such that the minimum load versus the peak RDG generation, therefore, it is desirable to tolerate large RDG integration at PCC and curtail it whenever there is voltage rise occurrence to certain set voltage range. The total amount of RDG curtailment annually can be determined by the no of occurrence of minimum load versus peak RDG generation. The only disadvantage of RDG curtailment is the reduction in the revenue which can affect the utility and the independent power producers because the electricity price is normally being influenced by the amount of load demanded. Logically, the loss of revenue would not be so much as the RDG curtailment will normally occur during the low load and RGD peak generation situation. Hence, the amount of curtail may be moderately low.

### Reactive power compensation strategy

Reactive power control is critical in the electrical grid to avoid voltage breakdown, voltage instability, and voltage rise when there is an unusual occurrence or eventualities at PCC. Voltage rise and instability occur in a system wherever there is insufficient of reactive power during RDG over generation, heavy loading and disturbances such as grid faults^50^. Installing Flexible Alternating Current Transmission System (FACTS) devices such as pulse width modulation distribution static compensator (PWMDSTATCOM) connected to the PCC of a large renewable farm, dynamic compensation of reactive power and voltage rise control capability can be realized since the lesser the voltage at PCC, the more the reactive power needed^51^. It is also very valuable in power system interruptions. Reactive power may be supplied by mechanisms embedded in the network itself and by additional elements inserted into the network to balance the reactive power of this system^52–54^. The FACT compensation method should be a requirement for a system with non-linear loads and a large RDGs integration to provide voltage support, attenuate voltage rise at PCC and stability in the event of network disturbance. If the voltage rise at PCC with a large RDGs integration is to be regulated, a compensator device should be installed at PCC^55^ which can be expressed from Eq. (10) to produce Eq. (22). The Eq. (22) can be re-expressed in Eq. (23) and the equation parameters are described in Abbreviation section.

22 $$\Delta V={V}_{RDG}-{V}_{a}\approx {R(P}_{RDG}-{P}_{L})+X(\,\pm\, {Q}_{c}-{Q}_{L}\,\pm\, {Q}_{RDG}),$$

where $\,\pm\, {Q}_{c}$ = Compensator reactive power (it can generate or absorb reactive power). The voltage rise can be easily controlled with reactive compensator. When the device is strategically installed at the PCC to generate or absorb reactive power, the voltage rise at that point would be considerate minimized, this could also allow more RDG penetration levels without the fear of PCC over voltage or voltage rise.

23 $$\Delta V={V}_{RDG}-{V}_{a}\approx R\left({P}_{RDG}-{P}_{L}\right)+X{Q}_{Import},$$

where $${Q}_{Import}=\,\pm\, {Q}_{c}-{Q}_{L}\,\pm\, {Q}_{RDG}.$$

If a critical situation is to be considered and the network operates at unity power factor, the Eq. (23) can be re-expressed in Eq. (24).

24 $$\Delta {V}_{Critical}\approx R{P}_{RDG\, Max}+X{Q}_{Import}.$$

RDGs always export active power $({+P}_{RDG})$. Thus, it may also export or import reactive power ${(\,\pm\, Q}_{RDG})$ depend on the RDG parameters e.g., synchronous generator can import power at a 0.96 power factor, whereas a wind turbine with uncompensated induction generator can import power at about a 0.9 power factor. Whereas the load consumes both active $({P}_{L})$ and reactive $({Q}_{L})$ power. The compensators may export or absorb reactive power $(\,\pm\, {Q}_{c})$ depend on the voltage rise occurrence at PCC. From the Eq. (24), it can be deduced that the increase in the amount of reactive power imported would bring the regulation of voltage rise at PCC. The higher the negative value of $(X{Q}_{Import})$, the lower the reduction in the voltage rise at PCC, this statement would be verified in the simulation carried out in the next section.

### Compensator modelling

**Figure 9:**
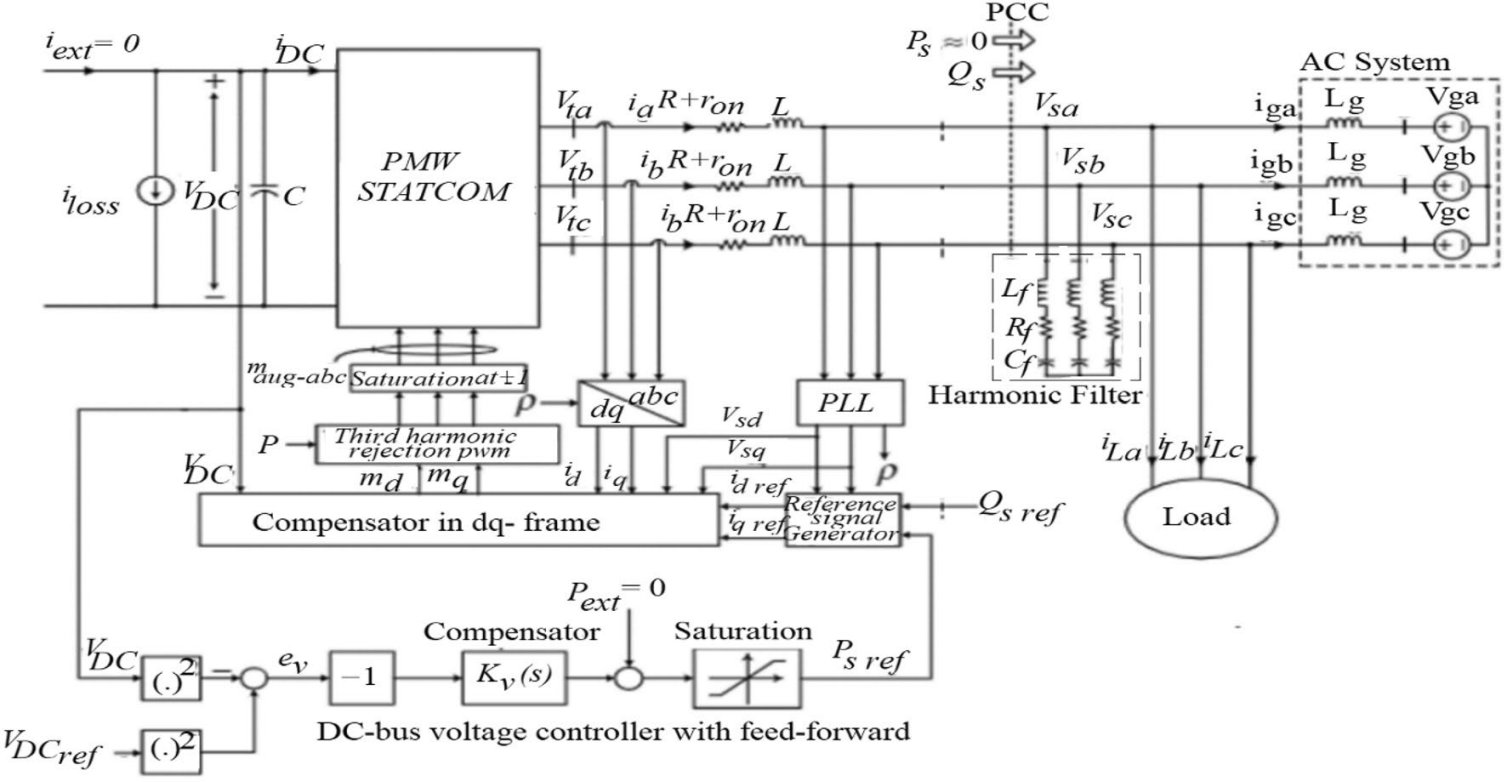
PWMDSTATCOM connection to PCC.

System compensation is carried out in this section to mitigate the voltage rise at the PCC with a large RDG integration to a distribution network. The pulse width modulation distribution static compensator (PWMDSTATCOM) is connected to the test system in Fig. 5 at PCC to mitigate the voltage rise by generation/absorption of reactive power to the system. PWMDSTATCOM parameters are grouped in two categories: Power and Control tab. Converter rating, current, nominal voltage, DC link voltage, impedance, and capacitance rating are specified in power tab while control tab consists of modes of operation (var and voltage control) and droop that control the slope (the regulator gains Kp and Ki). PWMDSTATCOM is a circuit-fed reactive power compensation device which is able to produce and/or absorb reactive power, such that the precise data of the grid is controlled to modify the output voltage^56^. It rectifies the direct current input (DC) voltage in an AC output voltage to compliment for the active and reactive power required by the system^57^. The PWMSTATCOM schematic representation and connection model to PCC is depicted in Fig. 9, 3-phase voltage source ${(V}_{ga}, {V}_{gb}, \, \text{and}\, {V}_{gc})$ represents an AC system which in series connection with a transmission line, $({L}_{g})$ represent inductance of the line while the resistance and transformer are assumed to be negligible. By controlling the real power of the system, $({V}_{dc})$ can be regulated. The $({V}_{dc})$ is supported by a direct current source which can be a DC energy source such as battery banks and $({P}_{s})$ compensates for the device power loss.

The electrical nodes on the PWMDSTATCOM connection to the 3-phase AC constitute PCC and the voltage at that point are $({V}_{sa}, {V}_{sb} \, \text{and}\, {V}_{sc})$. The phase lock loop PLL input is taken from PCC, and 3-phase loads are also supplied from the PCC. 3-phase RLC filters are connected in shunt with the PWMDSTATCOM at PCC to filter unwanted, current harmonics, amplitude and frequency signals of the load voltage from flowing into the grid. The PWMDSTATCOM has constant current characteristics when there is under-voltage/over-voltage, below/above the boundary, which permits PWMDSTATCOM to provide constant reactive power^57,58^. The relationship between the Alternating Current of the network voltage and the voltage at the PWMDSTATCOM alternating current side terminals offers the influence of reactive power flow. When the terminal voltage of the PWMDSTATCOM contact is above the network voltage, PWMDSTATCOM would inject reactive power to the grid and PWMSTATCOM act like a capacitor. As soon as the PWMDSTATCOM voltage is below the AC voltage, PWMDSTATCOM functions as an inductor and the reactive power flow is reversed, when the network voltage is equal to the PWMDSTATCOM voltage, there is no energy exchange^59,60^. The modelling of PWMDSTATCOM’s dynamic voltage regulation at PCC involves linearizing the non-linear circuit elements at the operating point of PWMDSTATCOM and when the behaviour becomes non-linear at the PCC. The modelling is divided into two, the first signal modelling is the PWMDSTATCOM’s dynamic voltage response which is obtained by applying a small (AC) signal on top of the DC operating points called large signal while the second modelling is to obtain DC operating point of PWMDSTATCOM at PCC called small signal.

### PWMDSTATCOM dynamic voltage (large signal)

The voltage at the PCC $({V}_{sa}, {V}_{sb} \, \text{and}\, {V}_{sc})$ are being regulated by PWMDSTATCOM with $({i}_{La}, {i}_{Lb }\, \text{and}\, {i}_{Lc})$ while $({i}_{a}, {i}_{b} \, \text{and}\, {i}_{c})$ are controlled. The relationship is presented in Eqs. (25)–(30), where $\left({V}_{null}\right)$ is the voltage of the AC system neutral point with respect to the midpoint of the VSC DC bus. When space phasor of balanced 3-three is considered, the sinusoidal function is produced in (31), $(\widehat{f})$, (θ_0_) and (*w*) are amplitude, phase angle and angular frequency. The sinusoidal function of the space phasor is given in Eq. (32), Eqs. (25) and (26) are multiplied both sides by the Eq. (32) and added to Eq. (33). Space phasor does not contain $({V}_{null})$, therefore, Eqs. (25)–(27) are added to produce Eq. (34).

25 $${V}_{sa}={L}_{g}\frac{{di}_{ga}}{dt}+{V}_{ga}+{V}_{null},$$

26 $${V}_{sb}={L}_{g}\frac{{di}_{gb}}{dt}+{V}_{gb}+{V}_{null},$$

27 $${V}_{sc}={L}_{g}\frac{{di}_{gc}}{dt}+{V}_{gc}+{V}_{null},$$

28 $${i}_{ga}={i}_{a}-{i}_{La},$$

29 $${i}_{gb}={i}_{b}-{i}_{Lb},$$

30 $${i}_{gc}={i}_{c}-{i}_{Lc},$$

$${f}_{{a}^{(t)}}=\widehat{f}\,\text{cos}\left(wt+{\theta }_{0}\right), {f}_{{b}^{(t)}}=\widehat{f}\,\text{cos}\left(wt+{\theta }_{0}-\frac{2\pi }{3}\right),$$

31 $${f}_{{c}^{(t)}}=\widehat{f}\,\text{cos}\left(wt+{\theta }_{0}-\frac{4\pi }{3}\right),$$

32 $$\vec{f}\left(t\right)=\frac{2}{3}\left({e}^{j0}{f}_{{a}^{(t)}}+{e}^{{j}^{\frac{2\pi }{3}}}{f}_{{b}^{(t)}}+{e}^{{j}^{\frac{4\pi }{3}}}{f}_{{c}^{(t)}}\right),$$

33 $$\vec{{V}_{s}}={L}_{g}\frac{\vec{d{i}_{g}}}{dt}+\vec{{V}_{g}},$$

34 $$\vec{{i}_{g}}=\vec{i}-\vec{{i}_{L}}.$$

Consider the AC voltage ${(V}_{ga}, {V}_{gb}, \, \text{and}\, {V}_{gc}):$

$${V}_{ga}={\widehat{V}}_{g}\,\text{cos}\left({w}_{{0}^{t}}+{\theta }_{0}\right), {V}_{gb}={\widehat{V}}_{g}\,\text{cos}\left({w}_{{0}^{t}}+{\theta }_{0}-\frac{2\pi }{3}\right),$$

35 $${V}_{gc}={\widehat{V}}_{g}\,\text{cos}\left({w}_{{0}^{t}}+{\theta }_{0}-\frac{4\pi }{3}\right).$$

When Eq. (35) is multiplied by Eq. (32), Eq. (36) is obtained. From Fig. 6, If $\left(dq\right)$ frame with angle $(p)$ is used to control PWMSTATCOM, Eq. (17) can be substituted for to give Eq. (37) similarly, Eq. (32) is substituted to give (35). Following the similar substitution in Eq. (34), Eqs. (39) and (40) are obtained. By comparison, the real and imaginary component can be obtained through derivative of Eq. (36) multiply by ${e}^{jp}$ to obtain Eqs. (41) and (42). Where $\left(w=\frac{dp}{dt}\right)$ and *(w)* is controlled by a phase lock loop (PLL) based on Eq. (43). The $[w\left(t\right)]$ in Eq. (43) represents a nonzero steady state value when $\left({V}_{sd}\right)$ settle at zero. Dynamic system is represented by Eqs. (39)–(43), where $\left({V}_{sq}\right)$ is the output, $\left({I}_{d}\right)$ and $\left({I}_{q}\right)$ are controlled input $\left({I}_{Id}\right)$ and $\left({I}_{Iq}\right)$ are disturbance inputs. Hence, the dynamic variable is $\left(w\right)$ depends on the operating point, but to further clarify the operating point, $\left({V}_{sq}\right)$ is substituted for in Eqs. (39) and (40) respectively. The dynamic responses of $\left(p\right)$ and $\left(w\right)$ are indicated in Eq. (44) where their natural and forced transient components are equal to zero.

36 $${\vec{V}}_{g}={\widehat{V}}_{{g}^{{e}^{j({w}_{{0}^{t+{\theta }_{0})}}}}},$$

$${\vec{V}}_{s}={V}_{{sdq}^{{e}^{jp}, }}{\vec{i}}_{g}={i}_{{gdq}^{{e}^{jp}}, } {\vec{V}}_{g}={\widehat{V}}_{{g}^{{e}^{j({w}_{{0}^{t+{\theta }_{0}}})}}},$$

37 $${V}_{{sdq}^{{e}^{jp}}}={L}_{g}\frac{d}{dt}\left({i}_{{gdq}^{{e}^{jp}}}\right)+{\widehat{V}}_{{g}^{{e}^{j({w}_{{0}^{t+{\theta }_{0}}})}}},$$

$${\vec{i}}_{g}={i}_{{gdq}^{{e}^{jp}}}, \vec{i}={i}_{{dq}^{{e}^{jp}}}, {\vec{i}}_{L}={i}_{{Ldq}^{{e}^{jp}},}$$

38 $${i}_{gdq}={i}_{dq}-{i}_{Ldq,}$$

39 $${i}_{gd}={i}_{d}-{i}_{Ld},$$

40 $${i}_{gq}={i}_{q}-{i}_{Lq},$$

41 $${V}_{sd}={L}_{g}\frac{{di}_{gd}}{dt}-{L}_{{g}^{{wi}_{gq}}}+{\widehat{V}}_{g}\,\text{cos}({w}_{{0}^{t}}+{\theta }_{0}-p),$$

42 $${V}_{sq}={L}_{g}\frac{{di}_{gq}}{dt}-{L}_{{g}^{{wi}_{gd}}}+{\widehat{V}}_{g}\,\text{sin}\left({w}_{{0}^{t}}+{\theta }_{0}-p\right),$$

43 $$\frac{dp}{dt}=w\left(t\right)=H\left(p\right){V}_{{sq}^{\left(t\right)}},$$

44 $$\frac{dp}{{dt}} = L_{g} H\left( p \right)\left( {\frac{{di_{gq} }}{dt} + wi_{gd} } \right) + \hat{V}_{g} H\left( p \right)\sin \left( {w_{{0^{t} }} + \theta_{0} - p} \right).$$

### PWMDSTATCOM dynamic voltage (small signal)

The dynamic voltage at PCC (small signal) can be obtained from the Eqs. (41)–(43) around a steady state operating point. Let perturbed variable be defined below, if $\tilde{p }/{p}_{0}\ll 1$, then Eq. (46) is obtained. From (46), perturbed variable is substituted equations in (48)–(50). Substitute for ${\widehat{V}}_{g}\,\text{cos}{p}_{0}$ and ${\widehat{V}}_{g}\,\text{cos}{p}_{0}$ in Eqs. (49) and (50) from (47) and (48). Similarly, perturbed of Eq. (45) is substituted for in Eqs. (43) and (44) and Eq. (50) is deduced. The Laplace transform of the Eqs. (49)–(51) produced Eqs. (52) and (53). The Eqs. (49)–(51) and its Laplace transforms (52)–(54) described a linear system that is the small signal equivalent of the system as described by the Eqs. (41)–(43). The dynamics of ${\widehat{V}}_{sd}(s)$ in terms of ${\widehat{I}}_{gd}(s)$ and ${\widehat{I}}_{gq}(s)$ can be obtained by elimination of ${\widehat{V}}_{sq}(s)$ in (38) and (39), then $\widehat{p}$ can be substituted in (37) thus, (40) is obtained. Where ${G}_{d}(s)$ and ${G}_{q}(s)$ are transfer function, which has parameters of ${I}_{gd}0$ and ${I}_{gq}0$. ${I}_{d}=0$, ${I}_{d0}={\widehat{I}}_{d}=0$, then the STATCOM exchange a small amount of real power with PCC, such that ${P}_{s}=0$ and the DC side power of ${P}_{loss}= {VDC}_{loss}$. Based on the Eqs. (39) and (40), Eqs. (56)–(59) are obtained. When ${\widehat{I}}_{gd}$ and ${\widehat{I}}_{gq}$ from the Eqs. (58) and (59) are substituted for in Eq. (55), the load and control effects are obtained in Eq. (60).

$${V}_{sd}={V}_{sd0}+{\tilde{V }}_{sd},$$

$${V}_{sq}=0+{\tilde{V }}_{sq},$$

$${i}_{gd}={i}_{gd0}+{i}_{gd},$$

45 $$w_{{0^{t} }} + \theta_{0} - p = - (p_{0} + \widetilde{p)} \Rightarrow \frac{dp}{{\underbrace {dt}_{w}}} = w_{0} + \frac{{\widetilde{dp}}}{{\underbrace {dt}_{w}}},$$

$$\,\text{cos}\left({p}_{0}+\tilde{p }\right)\approx \,\text{cos}{p}_{0}-\left(\,\text{sin}{p}_{0}\right)\tilde{p },$$

46 $$\,\text{sin}\left({p}_{0}+\tilde{p }\right)\approx \,\text{sin} {p}_{0}+\left(\,\text{cos} {p}_{0}\right)\tilde{p },$$

47 $${V}_{sd0}=-{L}_{{g}^{{w}_{0igd0}}}+{\widehat{V}}_{g}\,\text{cos}{p}_{0},$$

48 $$0={L}_{{g}^{{w}_{0igd0}}}-{\widehat{V}}_{g}\,\text{sin}{p}_{0},$$

49 $${\tilde{V }}_{sd}={L}_{g}\frac{{\tilde{di }}_{gd}}{dt}-{L}_{{g}^{wo{\tilde{i }}_{gq}}}-{L}_{{g}^{{i}_{gd0\tilde{w }}}}-\left({\tilde{V }}_{g}\,\text{sin}{p}_{0}\right)\tilde{p },$$

50 $${\tilde{V }}_{sq}={L}_{g}\frac{{\tilde{di }}_{gq}}{dt}-{L}_{{g}^{wo{\tilde{i }}_{gd}}}-{L}_{{g}^{{i}_{gd0\tilde{w }}}}-\left({\tilde{V }}_{g}\,\text{cos}{p}_{0}\right)\tilde{p },$$

51 $$\frac{\tilde{dp }}{dt}=\tilde{w }=H\left(p\right){\tilde{V }}_{sq},$$

52 $${\tilde{V }}_{{sd}^{(s)}}={L}_{{g}^{s\tilde{I }{gd}^{(s)}}}-{L}_{{g}^{w0\tilde{I }{gd}^{\left(s\right)}}}-{L}_{g}\left({i}_{{gq0}^{s}}+wo{i}_{gd0}\right)\tilde{p }\left(s\right),$$

53 $${\tilde{V }}_{{sq}^{(s)}}={L}_{{g}^{s\tilde{I }{gq}^{(s)}}}+{L}_{{g}^{w0\tilde{I }{gd}^{\left(s\right)}}}+{L}_{g}\left({i}_{{gd0}^{s}}+wo{i}_{gq0}\right)\tilde{p }\left(s\right),$$

54 $$\tilde{p }\left(s\right)=\frac{H(s)}{s}{\tilde{V }}_{{sq}^{(s)}},$$

55 $${\tilde{V }}_{{sd}^{(s)}}={G}_{{d}^{(s)}{\tilde{I }}_{{gd}^{(s)}}}+{G}_{{q}^{(s)}{\tilde{I }}_{{gd}^{(s)}}},$$

56 $${i}_{gd0}\approx -{i}_{Ld0},$$

57 $${i}_{gq0}={i}_{q0}-{i}_{Lq0},$$

58 $${\tilde{i }}_{gd}\approx -{\tilde{i }}_{Ld},$$

59 $$\tilde{i}_{gq} = \tilde{i}_{q} - \tilde{i}_{Lq} ,$$

60 $$\tilde{V}_{{sd^{\left( s \right)} }} = \underbrace {{ - G_{{d^{\left( s \right)} \tilde{I}_{{Ld^{\left( s \right)} }} - G_{{q^{\left( s \right)} \tilde{I}_{{Lq^{\left( s \right)} }} }} }} }}_{load \,effect} + \underbrace {{ - G_{{q^{\left( s \right)} \tilde{I}_{{q^{\left( s \right)} }} }} }}_{load \,effect}.$$

### Regulatory capability of PWMDSTATCOM

**Figure 10:**
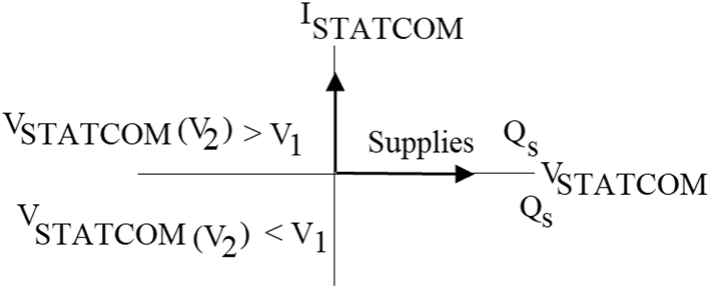
PWMDSTATCOM power operation.

**Figure 11:**
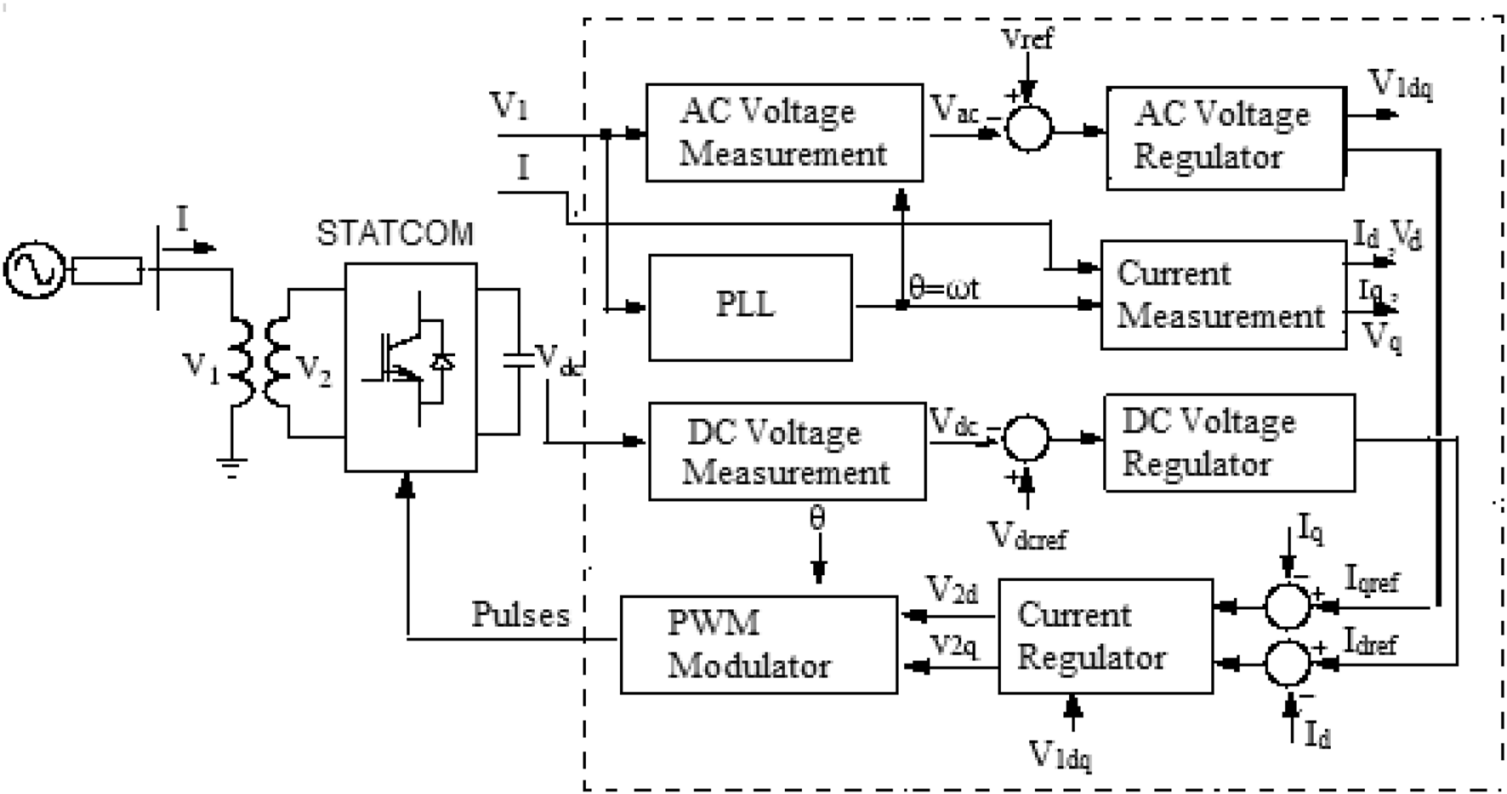
PWMDSTATICOM and its control system.

The PWMDSTATCOM regulation operation and the control diagram are depicted in Figs. 10 and 11^61^, where (V2) is the PWMDSTATCOM voltage and (V1) is the PCC voltage. If the voltage (V2) is lower than (V1), the current in the inductor is slightly displaced from the voltage (V1) produces an inductive current, then (Qs) becomes positive and PWMDSTATCOM absorbs reactive. When the voltage of the PWMDSTATCOM exceeds the PCC voltage, the current across the inductor is slightly offset from the voltage V1 that provides a capacitive current, then (Qs) is negative and the PWMDSTATCOM produces a reactive power. When the voltage of the PWMDSTATCOM is equal to the voltage of the PCC the current through the inductor is nil and consequently there is no power exchange^62^.

**Figure 12:**
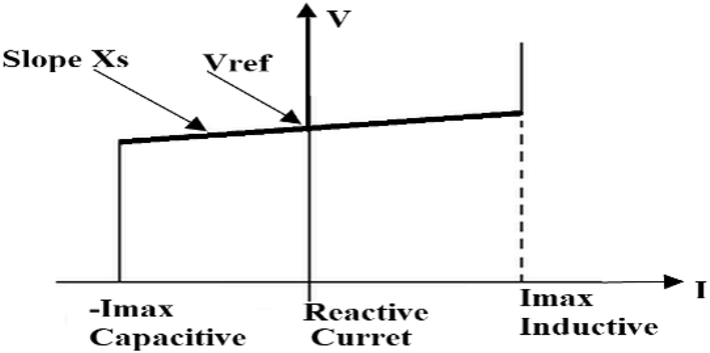
Voltage/current feature.

The 3-three voltage (V_1_) is matched by Phase Locked Loop (PLL), the direct axis and quadrature axis mechanisms of 3-three alternating current such as *(V*_*d*_*, V*_*q*_*, I*_*d*_*,* and *I*_*q*_*)* are configured by the output of the PLL (angle *θ* = *ωt*). The measured *(d)* and *(q)* mechanisms of (AC) positive sequence voltage/current, composed with (DC) voltage (*V*_*dc*_) are controlled. An outer regulation loop comprises of an AC voltage regulator and a DC voltage regulator. The reference current (*I*_*qref*_) for the current regulator is from the output of AC voltage regulator (*I*_*q*_ = current in quadrature with a voltage that controls reactive power flow). The reference current *(I*_*dref*_*)* for the current regulator is from the output of the DC voltage regulator (*I*_*d*_ = current in phase with voltage that controls the active power flow). An inner current control-loop system consisting of a current regulator/or controller, which control the direct current ($${I}_{d})$$, the quadrature current ($${I}_{q})$$, and the active and reactive power. The magnitude and phase of the voltage generated by the PWMDSTATCOM converter (*V*_2*d*_* V*_2*q*_) from the *(I*_*dref*_*)* are being controlled by current regulator, while DC voltage regulator and the AC voltage regulator (in voltage control mode) produces (*I*_*qref*_) reference currents. The direct power type regulator that predicts the voltage output, *V*_2_ *(V*_2*d*_* V*_2*q*_*)* from measurement *V*_1_ (*V*_1*d*_* V*_1*q*_) and the leakage reactivity of the transformer assist the current regulator. The change in reactive power is achieved by means of a voltage source converter connected to the secondary side of a coupler transformer. The voltage source converter utilizes forced-commutated power electronic devices (GTOs, IGBTs or IGCTs) to create a voltage (*V*_2_) from a DC voltage source. There are two ways in which PWMDSTATCOM can be used, VAR control and voltage regulation mode. When PWMDSTATCOM is in VAR control mode, its reactive power is maintained constant. In the voltage regulation mode, if the reactive current remains within the minimum current values (− Imax, Imax) imposed by the nominal value of the converter, the voltage is controlled to the reference voltage Vref. However, a voltage droop is usually used between 1 and 4% at maximum reactive power output and the V–I characteristic has the slope indicated in Fig. 12^63^ the V–I characteristic is stated in equation^60^. Inductive current is generated when current (I) is greater zero, while capacitive current is produced when the current generate is less than zero and reactive current when the current is zero as shown in Fig. 12.

61 $${\text{V}} = {\text{Vref }} + {\text{ Xs I}}{.}$$

By the adjustment of the phase angle and that of reference point, the bus voltage can be varied with the PWMDSTATCOM connected in parallel. When the grid voltage is on the high side or at the lower value over the boundary, PWMDSTATCOM behaves in its constant current features. It can produce reactive capacity at the boundary such as capacitive and inductive compensation and independently control its output current over the rated maximum capacitive or inductive range of the amount of AC system voltage.

### PCC voltage rise regulation

**Figure 13:**
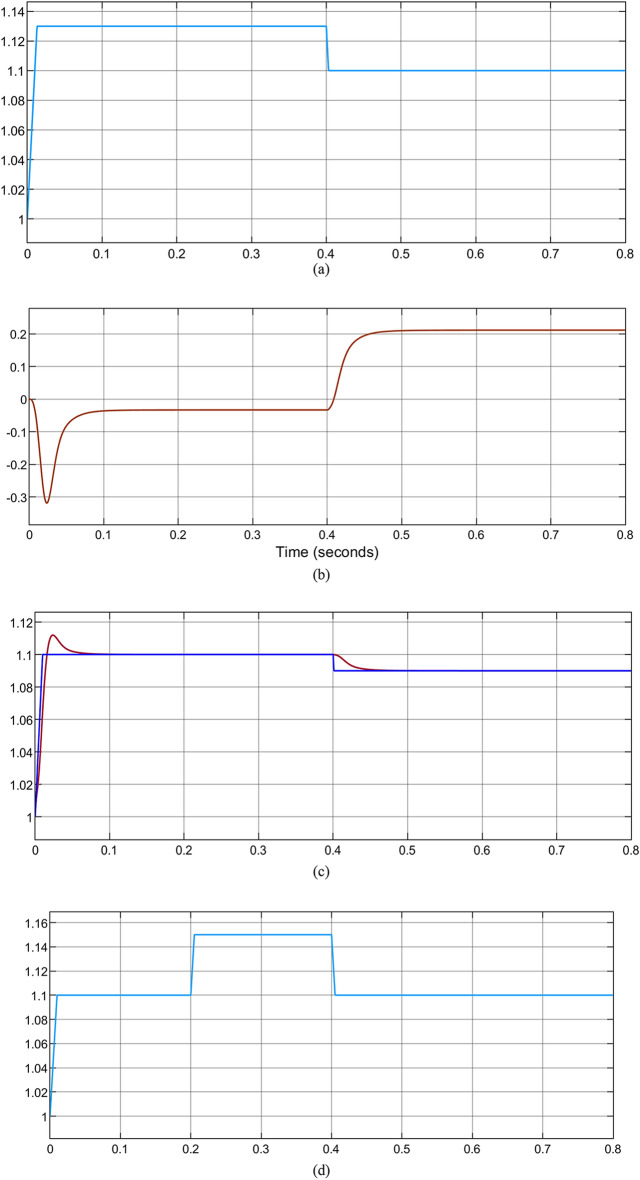

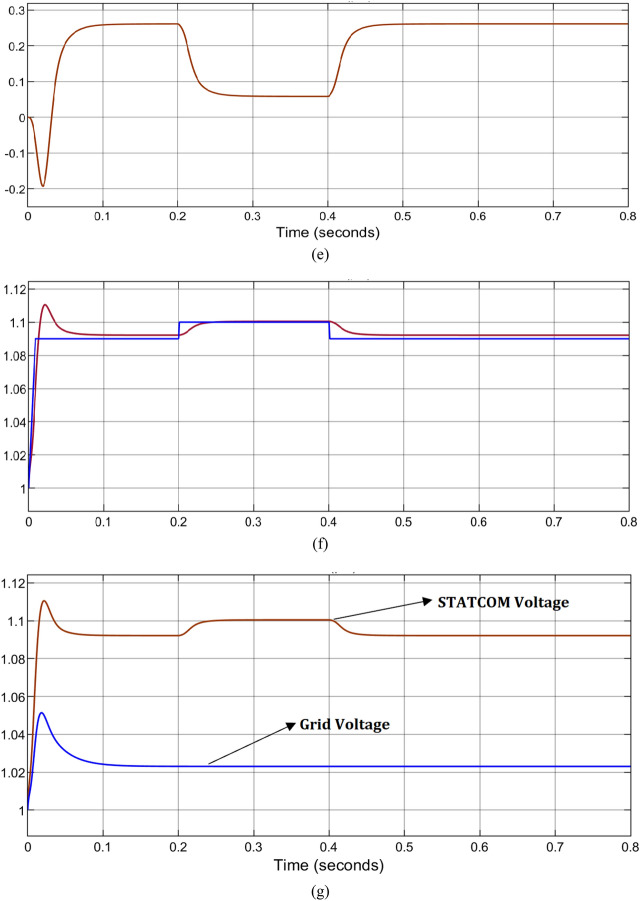
(**a**) Voltage rise at bus 684 with 80% RDG penetration, (**b**) reactive power generated by PWMDSTATCOM to minimize the voltage rise at PCC, (**c**) voltage rise regulation at PCC with PWMDSTATCOM, (**d**) voltage rise at bus 675 with 85% RDG penetration, (**e**) reactive power generated by PWMDSTATCOM to regulate voltage rise at PCC, (**f**) voltage regulation at PCC with PWMDSTATCOM and (**g**) grid voltage during PCC regulation.

By considering the electrical distribution network—with a large RDG penetration via PCC. In the case where the power penetration of RDG is 80% of its nominal power rating, there would be the occurrence of voltage rise at PCC. This must be regulated for the continuous operation of RDG as specified by IEEE 1457–2018 and South Africa grid codes. Otherwise, the RDG should be disconnected as per the IEEE 1457–2014 grid code. When the RDG is operating at 90% of its rated nominal power and injected to Bus 684, the results observed are shown in Fig. 13a–c. Voltage rise occurs up to 1.13 pu as depicted in Fig. 13a without PWMDSTATCOM connection to the system, which is not acceptable, the maximum permissible voltage at PCC is 1.1 pu as specified by the South Africa PCC voltage with RDGs integration. The operation of the PWMDSTATCOM is shown in Fig. 13b,c, during the voltage rise condition, the operating mode of the PWMSDTATCOM changes from the unity power factor to a voltage regulation mode to mitigate the voltage rise at the PCC to an acceptable range in relation to IEEE 1547 and South Africa grid code requirement. It is observed during the voltage regulation mode that the PWMDSTATCOM generates reactive power from − 0.3 kVAR to − 0.02 for the duration of 0.4 s to keep the PCC voltage to 1.1 pu and from − 0.02 kVAR to 0.2 kVAR at the duration of 0.4 s to 0.8 s as shown in Fig. 13b, more reactive power is being generated to the PCC by the PWMDSTATCOM, the less the occurrence of voltage rise and finally PCC voltage is sustained at 1.09 pu as shown in Fig. 13c between 0.4 and 0.8 s. Hence, the PCC voltage is within an acceptable range in agreement with IEEE 1547 and Southern Africa grid code requirement. The network takes a more RDG penetration level up to 120% with voltage regulation at PCC without violating the grid code act unlike when grid code is violated at 80% penetration without voltage regulation at PCC. Figure 13d–f shows the simulation results when PCC is changed from bus 684 to bus 675 to observe the impact of RDG. Voltage rise occurs between 0.2 and 0.4 s up to 1.15 pu with 85% RDG penetration levels as shown in Fig. 13d, but reactive power is generated by the PWMDSTATCOM to the PCC to keep the voltage within an acceptable level as shown in Fig. 13e. The PWMDSTATCOM maintains the PCC voltage at 1.1 pu as shown in Fig. 13f while 130% penetration levels are achieved without grid code violation unlike the occurrence of voltage rise at 85% penetration level without PCC regulation. The voltage that flows across the grid is 1.021 pu as shown in Fig. 13g which is within an acceptable range.

## Conclusion

The mathematical model of an electrical distribution network with an RDG—penetration has been developed in the paper. The developed model has further been used to investigate the effect of voltage rise and reverse power flow on an electrical distribution network. An advance controller with PWM control-algorithm has also been used to normalize the voltage rise at PCC and to mitigate the reverse power flow problem when operating at a worst critical scenario of minimum load and maximum power output from RDG. Initially, the simulations carried out in the paper has shown that the integration of RDG into a distribution network improve the voltage profile of the power system. However, when a large RDG is considered for integration, there would be a potential voltage rise threat at the PCC. For this scenario, the utility or independent power producers should consider installing an advanced controller at PCC, e.g., the PWM based DSTATCOM, for voltage rise regulation. To further improve the power quality of a large RDG integration at PCC, as a future research direction, online communication model for monitoring of voltage rise when it is out of limit can be considered for continuous measurement of voltages, currents and phase shifts using voltage quality analyzers. An automation system with voltage sensor can be developed, such that it depends on the generation from the RDG facility and the grid voltage for imbalance of electrical consumers at different phases.

## Appendix 1

**Table:**

Bus A	Bus B	Active power (KW)	Reactive power (kVAr)	Line length /km	Resistance and reactance/km
632	645	0	0	0	0.625 + j0.3125
632	633	35	6	2	0.625 + j0.3125
633	634	35	6	2	0.625 + j0.3125
645	646	35	6	2	0.625 + j0.3125
650	632	35	6	2	0.625 + j0.3125
684	652	35	6	2	0.625 + j0.3125
632	671	35	6	2	0.625 + j0.3125
671	684	35	6	2	0.625 + j0.3125
671	680	35	6	2	0.625 + j0.3125
671	692	35	6	2	0.625 + j0.3125
684	611	35	6	2	0.625 + j0.3125
692	675	50	20	5	0.625 + j0.3125

## List of symbols

$\hat{V}_{a}$: Sending end voltage
$\hat{V}_{b}$: Receiving end voltage
R: Resistance of the line
X: Reactance of the line
$\widehat{I}$: Current flows through the line
P: Real power flowing through the network to the load
Q: Reactive power flowing through the network to the load
$P_{Load}$: Real power of the load
$Q_{Load}$: Reactive power of the load
$P_{RDG}$: Renewable distributed generation active power
$Q_{RDG}$: Renewable distributed generation reactive power
*P*_*L*_: Active power of the load
*Q*_*L*_: Reactive power of the load
$R + jX$: Impedance of the line
$\left( {\hat{f}} \right)$: Amplitude of the line to neutral
$$w_{0}$$: AC frequency
$$\theta_{0}$$: Phase angle
(*V*_*null*_): Voltage of the AC system neutral point
(*V*_*sq*_): Voltage output
(*I*_*d*_) and (*I*_*q*_): Control input
(*I*_*Id*_) and (*I*_*Iq*_): Disturbance inputs
V: Positive voltage sequence
Vref: Reference voltage
Xs: Slope/droop resistance
I: Reactive current
I > 0: Inductive current
